# A comprehensive database of squirrel distribution and occurrence in South Asia

**DOI:** 10.3897/BDJ.11.e109946

**Published:** 2023-10-27

**Authors:** Udayraj Swati, Senan D'Souza, Palassery Suresh Aravind, Rakesh Kumar Muni, Nandini Rajamani

**Affiliations:** 1 IISER Tirupati, Tirupati, India IISER Tirupati Tirupati India

**Keywords:** database, occurrence, social media, citizen science, museum records, squirrels, South Asia, Asia

## Abstract

**Background:**

The Squirrels of South Asia (SOSA) database compiles comprehensive distribution and occurrence information on all squirrel species that occur in this region (34 species). These 34 squirrel species, including tree, flying and ground squirrels, represent 14% of global sciurid diversity. The database collates curated data from various sources such as museums, literature, primary fieldwork, citizen science and social media platforms and covers the entire distributional ranges of the target species, including countries in Central Asia and Southeast Asia when required. The SOSA database enhances our understanding of squirrel distribution, population dynamics and their conservation needs in South Asia by consolidating information. It aims to be a valuable resource for researchers, conservationists and wildlife enthusiasts.

**New information:**

As of March 2023, the database comprises over 40,000 records of 34 species in over 30 countries globally. Spending an average of 334 hours on each species, more than 20 data collectors put in over 10,000 hours to gather, curate and build this database. The database has resulted in novel records of species occurrence in regions and countries that are poorly represented in currently available global data repositories. The current version which has been made public via GBIF comprises of 1187 records of all 34 species across multiple sources. This is a subset of the SOSA database.

## Introduction

South Asia is home to several species of squirrels that occur across the Palearctic, Oriental and Indo-Malayan Regions (Datta and Nandini 2014). Due to rising deforestation and urbanisation, South Asian forests, one of the most biologically diverse ecosystems on the planet, are being destroyed (Hasnat et al. 2018). The small mammals in this region are under severe threat from several factors, like habitat degradation and hunting for the illicit wildlife trade (Willcox 2020).

Thirty-four species of squirrels occur in the South Asian region (India, Afghanistan, Bnagladesh, Bhutan, Maldives, Nepal, Pakistan and Sri Lanka), comprising 14% of the global diversity of squirrels (Whatton et al. 2012). Squirrels, like most other small mammals, have not been well studied in South Asia, which hampers our ability to prioritise endangered taxa and place them at the forefront of research on species declines (McKinney 1999, Koprowski and Nandini 2008). Squirrels can be broadly divided into three categories, based on their diversity in lifestyle, habits and morphology - tree squirrels, flying squirrels and ground squirrels. They play vital ecological functions like seed dispersal, pollination and regulating plant growth (Lacher et al. 2019). They also serve as a significant prey base in the landscapes where they occur (Byholm et al. 2012). Squirrels are susceptible to environmental pressures, such as urbanisation, habitat modification and climate change and can serve as model study systems (Sol et al. 2013). The first step in understanding how species respond to such changes is to determine their geographic range by documenting where they occur.

Databases on species occurrence are crucial for understanding biodiversity dynamics because they provide a comprehensive record of when and where a species has been observed (Chapman 2005). This information aids in conservation efforts, helps identify areas of high biodiversity and enables the study of species' responses to environmental changes (Jetz et al. 2019). Due to technical improvements and the emergence of online portals like the Global Biodiversity Information Facility (GBIF), which combine occurrence information from various datasets, the number and accessibility of data on species occurrences have significantly risen in recent years (Petersen et al. 2021). Managers of biodiversity and applied conservation practitioners use such data to understand species occurrence trends and update/infer the IUCN status of species periodically (Bloom et al. 2017). For example, Species Distribution Models (SDMs) are frequently used to estimate how climate change affects species range shifts and to understand environmental limitations on range expansion and contraction (Jetz et al. 2019).

The rapid spread of internet connectivity and access to mobile technology enables the interested public to capture and share information and simultaneously allows researchers to serendipitously access large-scale secondary data in ways that were not possible a couple of decades ago (Pocock et al. 2017). The objective of creating the Squirrels of South Asia database is to compile comprehensive and up-to-date information on squirrel occurrence across South Asia by leveraging a diverse range of data sources, including traditional data, citizen-science contributions and social media data. This database aims to serve as a valuable resource for researchers, conservationists and wildlife enthusiasts, providing a centralised platform to access, analyse and contribute to the understanding of squirrel species in Asia.

## Sampling methods

### Sampling description

**Data Collection**

We collected data from three major sources. Traditional data sources included museum data, information from literature and primary field data. The second source of data included records from citizen-science platforms (five platforms) and the third source of data encompassed social media platforms (14 sources) (details in Table 1).

A pipeline was created for the data collection and all volunteers (15) on the project were trained to follow this to maintain consistency in data collection. For all data records, irrespective of source, we noted essential information like species name, location, date/year of observation and observer/author. Media data were examined/downloaded when available. In addition to this, all other relevant data associated with each record were noted (e.g. behaviour, ecological information etc.). Details of how data records were collected within each source are listed below.

**Traditional data**

Museum data: Museum data were collected from the GBIF (https://www.gbif.org/) portal, which collated data from 40 museums across the world (Table 2). In addition to this, specimen data from the Bombay Natural History Society (BNHS) Museum and the Zoological Survey of India (ZSI) were included. Specimen data from BNHS were included when data were collected as part of other projects in the research lab and, hence, this is not a comprehensive list of their squirrel collections. Data on specimens in the ZSI collections were obtained through literature searches, detailed below. Sometimes, publications of ZSI included details of species occurrence through a listing of specimens collected during field expeditions. However, on other occasions, ZSI publications listed occurrence data in compilations and geographic summary reports, where specimens were not explicitly collected/listed. We include both sources as ZSI records.

**Literature data**:

A two-stage approach was used to find traditional sources that listed squirrel occurrence across the target region. The first stage included searching for species occurrence records on two online data engines, Google Scholar and Biodiversity Heritage Library, using species' scientific names only. Only current genera and species names were used for these searches. The Biodiversity Heritage Library (BHL) is a repository of old documents, books and natural history notes that are not always indexed by search engines like Google Scholar. BHL was specifically searched to add records from colonial expeditions from 1800 onwards. Google Scholar was used as the primary search engine as it has substantially higher coverage and indexing of academic literature than traditional search engines (Martín-Martín et al. 2018). When search hits were found on Google Scholar, a snowball search approach was used to locate the original references (Wohlin 2014). Only original references of species occurrence were used to build the database.

Two years into the building of the database, all curated location points were plotted on a map and gap regions with no records were identified. For instance, large parts of Central Indian states like Madhya Pradesh, Chhattisgarh, Odisha and Jharkhand etc., were lacking in records. However, we know from personal communications/experience that squirrels occur here. In such cases, a second search was performed on Google Scholar using a combination of search terms, including State names (old and new), District names, names of protected areas and species common names to find more specific references from outside mainstream publication indexes. These were usually grey literature (occurrence information outside traditional literature) like survey reports or Forest Department documents. All of these were added to the database and Suppl. material 1 has the details all the references examined species-wise.

Primary field data: Field survey data included occurrence records collected directly from the field by researchers within the lab (20 lab members) who have been trained to identify squirrel species by sight and call. Typically, the training includes examination and cross-validation of species identity using known photographs and calls and discussions during shared field observations.

**Citizen-Science data**

Across all citizen-science portals, searches were conducted using the scientific names of squirrel species. We did not use common names for searches, as all the citizen-science portals identify data objects by their scientific names. iNaturalist (https://www.inaturalist.org) is a global online citizen-science platform that crowdsources biodiversity information and organism occurrence using automated and curator-led species identification (Nugent 2018). The website allows search-based downloads of bulk records, but does not automatically download media (calls, videos and images). The URL links of image files associated with each record were then used to import the images into our datasheets. The media records were verified for species identification during curation for records with calls and videos, but these files were not imported into our datasets. All records for a species were downloaded irrespective of the quality grade assigned by the curating protocol followed by iNaturalist. Project Noah (https://www.projectnoah.org/) is a global citizen-science platform where naturalists and nature enthusiasts upload images of organisms. The squirrel records were directly downloaded from the Project Squirrel mission page for this platform. The species of interest were later filtered out from the downloaded data and the images were imported into our Airtable datasheets (a spreadsheet-database hybrid tool) using the image URL links. Two India-specific citizen-science platforms were explored, given their popularity with Indian naturalists; the taxa-agnostic India Biodiversity Portal (https://indiabiodiversity.org/) and the mammal-specific Mammals of India (https://www.mammalsofindia.org/). India Biodiversity Portal is a repository of biodiversity information designed to collate information about the biodiversity of the Indian subcontinent (Vattakaven et al. 2016). Mammals of India is a peer-reviewed web-based repository of Indian mammals (Bayani et al. 2023). Squirrel records from the India Biodiversity Portal and Mammals of India platforms were downloaded from GBIF using species' scientific names.

Interested naturalists, students and researchers at or affiliated with IISER Tirupati were invited to join a campus-wide WhatsApp group dedicated to sharing information about squirrels in one’s surroundings. Members post photographs, calls, videos and sighting locations (phone-based location within WhatsApp) and curators identify species and engage with group members on aspects of squirrel behaviour and ecology. This initiative is listed as a citizen-science source rather than a social media source, as data are collected and curated on a specific project-linked platform.

**Social media data**

On all social media platforms (Table 1), species-wise searches were performed using common and scientific names for each species. Most users are not naturalists with comprehensive knowledge of biodiversity and we used combinations of English common and local language names to extract records. For social media data, only search results with media files (images, audio, video) that allowed confirmation of species identity were included in the database. For all search results, the associated post data, including species identification given by the user, user name, date of posting, date of observation and location information, were noted. In some instances where crucial information like location was missing, the observer was contacted for it and the information was later noted down. Researchers manually entered each entry into the datasheets (17 people over two years, including volunteer interns).

Most platforms allowed media (images, audio, video) associated with posts to be downloaded directly (Facebook, Minden Pictures, India Nature Watch, Twitter, iStock, 500pix, Nature Picture Library, India Wilds and Wikimedia). In cases of platforms (e.g. Instagram and Pinterest) that did not allow direct downloads of images, we accessed the images stripped of metadata. An R script was used to download images with metadata from Flickr, which was uploaded manually to the Airtable datasheet. All images were then manually uploaded to the respective data record.

### Quality control

**Data Curation**

All records, irrespective of their source, were curated for confirming species identification and determining location. Given low public awareness of squirrel species, we expected high error rates with contributors' assignment of species identity. A key of species images and calls was used to maintain consistency across the curatorial team. To ensure verification of species identity, media (photographs, audio and video data) were collected when possible or cross-checked on the source site. Each record was manually verified for species identity by one or two of four curators (more curators for less-familiar species). A curator manually eliminated duplicate records by comparing species-specific data across several sources. An internal history of deleted duplicate records was maintained. Subspecies level information in all records was collapsed to the species level. If only genus-level information were available, the record was discarded unless there were accompanying media to confirm species identification. Social media records/posts that did not have the required media to confirm species identity were discarded.

The location for each entry was also curated carefully and the location was parsed into four spatial categories - specific location, broad area, state/province and country. Specific location attributes to records where the exact location was mentioned are unique and have high spatial accuracy. The broad area is attributed to a larger place like a city or a town. The state/province refers to a larger administrative unit in which the broad area is located. The country is attributed to a major administrative unit in which the location occurs. For example, suppose a squirrel was recorded in the Victoria Memorial in Kolkata. In that case, the location details are as follows - specific location: Victoria Memorial, broad area: Kolkata, state/province: West Bengal, country: India. If the observer specified only a larger generalised area, like a range within a protected area, then we considered that as a broad area and not a specific location. A georeference was added when unavailable in the original post. All location data were imported into Google Sheets and the map tool Geocode by Awesome Table was used to obtain latitude and longitude data for places. Curators often contacted observers on social media platforms to confirm details before an entry was finalised.

**Database Structure**

A combination of .csv files, Google Sheets, Google Drive and Airtable were used to collect and curate the records. Once the curation was complete, all data were migrated into a database following the Darwin Core Standard for data representation. Species taxonomy followed international nomenclature following the IUCN taxonomy, consistent with the GBIF Backbone Taxonomy (GBIF Secretariat 2011, International Union for Conservation of Nature 2022) . Data will be uploaded to public platforms, specifically to GBIF, in batches over the coming years.

A preliminary version of the database with 1187 curated records (Swati et al. 2023) has been made public on the GBIF portal.

## Geographic coverage

### Description

**Figure 1.**
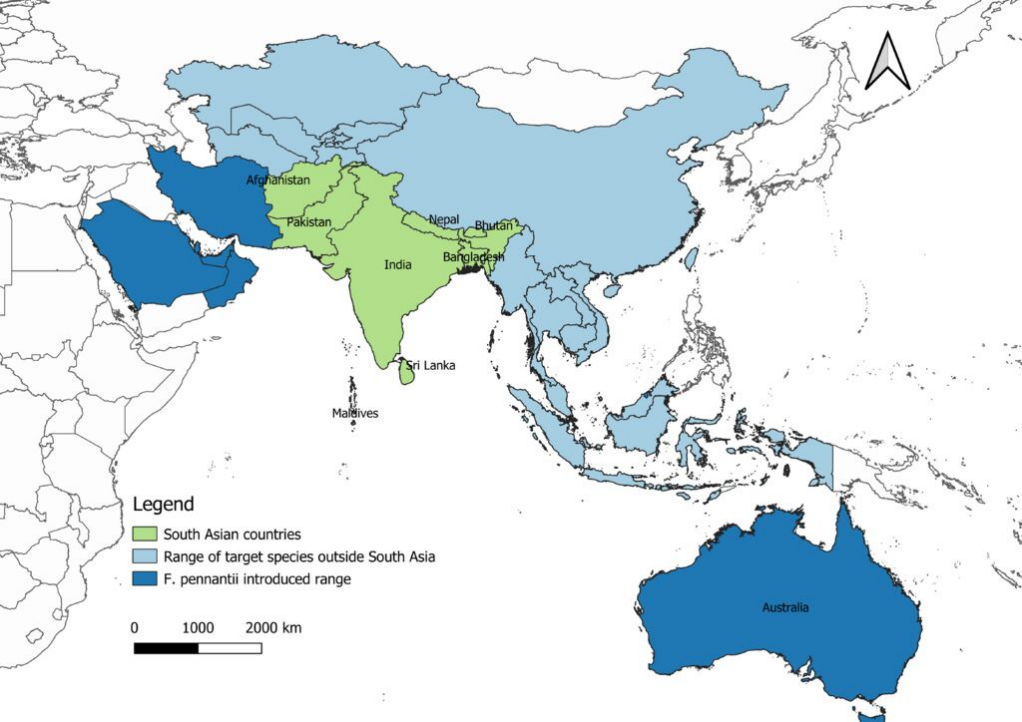
Map showing countries from which South Asian squirrel occurrence records have been collected.

For this database, we collected comprehensive occurrence records across the entire geographic ranges of the target species (Fig. 1). Species geographic range maps from the IUCN Red List of Threatened Species (IUCN 2022) were used to determine the final geographic ranges of species after consulting other sources (Whatton et al. 2012, Datta and Nandini 2014) to rule out conflicting range information. The geographic distribution of 16 species of squirrels found in South Asia extends into East and Southeast Asia, while the ranges of 12 species of squirrels are restricted to South Asia (*Biswamoyopterusbiswasi*, *Eoglaucomysfimbriatus*, *Eupetauruscinereus*, *Funambuluspalmarum*, *Funambulussublineatus*, *Funambulustristriatus*, *Petinomysfuscocapillus*, *Ratufaindica*, *Ratufamacroura*, *Funambuluslayardi*, *Funambulusobscurus*). Six species of squirrels, including two species in Afghanistan, have ranges extending to Central Asia and the Palearctic. One species of palm squirrel (*Funambuluspennantii*) has been introduced to the Middle-East and Australia, well outside its native geographic range. When squirrel species in this database were found to occur in countries outside of South Asia (e.g. Central/East/Southeast Asia), their occurrence data were collected across all countries in their geographic ranges, including the introduced/invaded regions (for *F.pennantii*) (Csurhes 2016, Victor and Cuellar Soto 2020). Thus, this database is currently a comprehensive set of occurrence records for all South Asian squirrel species, even when the species found here are not restricted to this region alone.

## Taxonomic coverage

### Description

We created a list of 34 species of squirrels that occur in South Asia after assessing Squirrels of the World (Whatton et al. 2012), Mammals of South Asia (Datta and Nandini 2014), GBIF Backbone Taxonomy (GBIF Secretariat 2011), the IUCN Red List (International Union for Conservation of Nature 2022) and the Mammal Diversity database (Burgin et al. 2018). Species that were listed as occurring in South Asia, but for which we could not find any records, are not listed in this current database. For instance, Burgin et al. (2018) list *Petauristaalborufus* as occurring in India, but we could find any records of any kind for the same. The final species list in this paper followed the GBIF Backbone Taxonomy and used it as a guiding framework (GBIF Secretariat 2011). An earlier version of this database focused only on species in India (Udayraj et al. 2022), which was subsequently expanded to the current database. Future versions and uploads of the database will be updated as and when we find records for previously unrecorded species in the region.

## Temporal coverage

**Data range:** 1766-1-01 – 2022-12-31.

### Notes

The earliest records of the presence and occurrence of squirrel species with accurate geolocation information date back to the late 1700s. For this database, occurrence data were systematically collected for the period spanning over two centuries until May 2023. The time frames for traditional data are from 1766 to 2023; citizen-science data from 1998 to 2023; and social media data from 2005 to 2023.

## Usage licence

### Usage licence

Creative Commons Public Domain Waiver (CC-Zero)

## Data resources

### Data package title

IISERTPT Squirrels of South Asia Database - Version 1

### Resource link

https://doi.org/10.15468/mnqzjv

### Alternative identifiers

https://www.gbif.org/dataset/f842ea5a-e38c-4be2-924d-c065b41215b0

### Number of data sets

1

### Data set 1.

#### Data set name

Squirrels of South Asia Database - Version1

#### Description

The table contains details of the column labels and its description of the GBIF dataset

**Data set 1.** 

Column label	Column description
id	Globally unique id.
basisOfRecord	The specific nature of the data record.
associatedReferences	A list (concatenated and separated) of identifiers (publication, bibliographic reference, global unique identifier, URI) of literature associated with the dwc:Occurrence.
occurrenceID	Globally unique identifier.
recordNumber	An identifier given to the Occurrence at the time it was recorded.
year	The four-digit year in which the Event occurred, according to the Common Era Calendar.
month	The integer month in which the Event occurred.
day	The integer day of the month on which the Event occurred.
continent	The name of the continent in which the Location occurs.
country	The name of the country or major administrative unit in which the Location occurs.
countryCode	The standard code for the country in which the Location occurs.
stateProvince	The name of the next smaller administrative region than country (state, province, canton, department, region etc.) in which the Location occurs.
locality	The specific description of the place.
decimalLatitude	The geographic latitude (in decimal degrees, using the spatial reference system given in geodeticDatum) of the geographic centre of a Location.
decimalLongitude	The geographic longitude (in decimal degrees, using the spatial reference system given in geodeticDatum) of the geographic centre of a Location.
coordinateUncertaintyInMetres	The horizontal distance (in metres) from the given decimalLatitude and decimalLongitude describing the smallest circle containing the whole of the Location.
geodeticDatum	The ellipsoid, geodetic datum or spatial reference system (SRS), upon which the geographic coordinates given in dwc:decimalLatitude and dwc:decimalLongitude are based.
scientificName	The full scientific name, with authorship and date information, if known.
kingdom	The full scientific name of the kingdom in which the taxon is classified.
phylum	The full scientific name of the phylum or division in which the taxon is classified.
class	The full scientific name of the class in which the taxon is classified.
order	The full scientific name of the order in which the taxon is classified.
family	The full scientific name of the family in which the taxon is classified.
genus	The full scientific name of the genus in which the taxon is classified.
specificEpithet	The name of the first or species epithet of the scientificName.
vernacularName	A common or vernacular name.
nomenclaturalCode	The nomenclatural code (or codes in the case of an ambiregnal name) under which the scientificName is constructed.
taxonRank	The taxonomic rank of the most specific name in the scientificName.
dynamicProperties	A list of additional measurements, facts, characteristics or assertions about the record. Meant to provide a mechanism for structured content. In this IUCN status has been added for each species.

## Additional information

### Discussion

The use of social media platforms is increasing exponentially across India and South Asia, yet these still need to be explored as sources for harvesting biodiversity information. While studies are increasingly using social media and citizen-science groups to harvest biodiversity data (Marcenò et al. 2021, Barman et al. 2022, O'Neill et al. 2023), our study is unique in a few respects.

Most studies typically collate data from taxon-specific, location-specific or event-specific groups on social media platforms like Facebook or citizen-science portals like iNaturalist. Such groups typically have curators who perform the first line of filtering and identification. Our study not only gathers data across multiple platforms, but employs naive searches to collate information across each platform/website. This implies that we redirect significant efforts towards data curation, as every point has to be validated by our internal team. Many records are also verified by dialogue and direct engagement with observers. This amounts to many work hours towards building a highly accurate and reliable database. Time and effort spent towards obtaining data were variable across the 23 data sources explored and database building up until March 2023 involved the efforts of four curators and 15 interns to collect over 40,000 records. The total time invested is upwards of 10,000 hours, with an average of 334 hours spent on each species.

We also propose to continue data collection from these sources over the next few years to add records till we reach saturation of records for species across their distribution ranges. For specific regions and species, we will invest in targeted efforts through workshops and citizen drives, as passive data collection might not be the ideal strategy. To date, we have launched a website to enable identification and education about squirrels in India (https://squirrelsofindia.org) and employ accompanying social media handles (https://www.facebook.com/groups/412739976780858, https://www.instagram.com/squirrels.of.india/) to encourage participation and dialogue. Versions of the database will be uploaded on public platforms, especially GBIF, on a regular basis.

We believe that building this database is a significant contribution to the field of mammalogy, particularly small mammal research in Asia. By consolidating and organising comprehensive information on squirrels inhabiting the South Asian Region, this database offers numerous benefits in terms of scope and impact. In terms of scope, the database provides valuable data to researchers, conservationists and policy-makers, aiding in a better understanding of the diversity and dynamics of the squirrel populations in this region (Cabrelli et al. 2014, Darrah et al. 2017, Pelletier et al. 2018). Additionally, this database goes beyond mere compilation of existing information. It also serves as a platform for data integration, enabling researchers to contribute new findings and update existing records through direct engagement. This approach fosters a continuous expansion of knowledge, allowing for the inclusion of new species discoveries, range extensions and updated conservation assessments. Consequently, we propose that the database will become a tool that adapts to the evolving understanding of South Asian squirrel biodiversity.

## Supplementary Material

48FC62BA-F268-5D21-955E-216A0A33B391 10.3897/BDJ.11.e109946.suppl1 Supplementary material 1 List of literature data from which occurrence data has been included in the database Data type PDF file File: oo_881392.pdf https://binary.pensoft.net/file/881392 Udayraj Swati, Senan D'Souza, Palassery Suresh Aravind, Rakesh Kumar Muni, Nandini Rajamani

## Figures and Tables

**Table 1.** Details of the three major sources of occurrence data.

**Traditional Data Sources**	**Citizen-Science Data Sources**	**Social Media Data Sources**
Museum data, Google search for literature, Biodiversity Heritage Library, Primary field data	iNaturalist, Mammals of India, India Biodiversity Portal, Project Noah	Instagram, Facebook, Twitter, YouTube, Flickr, 500px, Minden Pictures, Nature Picture Library, Alamy, iStock, India Nature Watch, Wikimedia, Pinterest, India Wilds

**Table 2.** Species list and the museums from which specimen data have been included in the database. Species and genus names used were in accordance with the listing on the GBIF Backbone Taxonomy. Museum Codes (Institution codes) and Names were used in accordance with the Global Registry of Scientific Collections (GRSciColl) and the Darwin Core Archive data standard (legend below table).

Squirrel species	Museums
*Belomyspearsonii* (Gray, 1842)	AMNH, FMNH, BYU, MNHN, NHMUK, NSMT, SMF, USNM, Naturalis
*Biswamoyopterusbiswasi* Saha, 1981	ZSIC
*Callosciuruserythraeus* (Pallas, 1779)	AMNH, ASNHC, BPBM, CAS, FMNH, KU, LACM, MACN, MCZ, MHNG, MNHN, MVZ, MZLU/LUND, NHMO/UiO, NHMUK, NML, NMR, NSMT, RBINS, ROM, SMF,UAM, UF, UMZM, USNM, UWBM, ZFMK, ZMH, YPM
*Callosciuruspygerythrus* (I. Geoffroy Saint-Hilaire, 1833)	AMNH, FMNH, LACM, MCZ, MNHN, NHMO/UiO, PSM, SMF,UMZM, USNM, YPM, ZFMK, ZMH
*Dremomyslokriah* (Hodgson, 1836)	AMNH, FMNH, KU, MCZ, MNHN, NHMUK, SMF, TCWC, UMZM, USNM, YPM, ZMH, Naturalis
*Dremomyspernyi* (Milne-Edwards, 1867)	AMNH, BYU, FMNH, KU, MCZ, MNHN, MVZ, NHMUK, SMF, TCWC, UMZM, USNM, ZFMK, Naturalis
*Dremomysrufigenis* (Blanford, 1878)	AMNH, FMNH, MCZ, MHNG, MNHN, MVZ, NHMUK, NSMT, RBINS, ROM, SMF, UMZM, USNM
*Eoglaucomysfimbriatus* (Gray, 1837)	nil
*Eupetauruscinereus* Thomas, 1888	Naturalis
*Funambuluslayardi* (Blyth, 1849)	MHNG, FMNH, NHMUK, USNM
*Funambulusobscurus* (Pelzeln & Kohl, 1886)	nil
Funambulus (Funambulus) palmarum (Linnaeus, 1766)	Naturalis, BNHS, ZSIC, NHMUK, AMNH
Funambulus (Prasadsciurus) pennantii Wroughton, 1905	Naturalis, BNHS, ZSIC, AMNH
Funambulus (Funambulus) sublineatus (Waterhouse, 1838)	Naturalis, NHMUK
Funambulus (Funambulus) tristriatus (Waterhouse, 1837)	AMNH, FMNH, MCZ, MHNG, NHMO/UiO, NMBT, ROM, ZFMK, ZMH
*Hylopetesalboniger* (Hodgson, 1836)	Naturalis
*Hylopetesphayrei* (Blyth, 1859)	NSMT
Marmota (Marmota) caudata (Geoffroy, 1844)	ZMMU
Marmota (Marmota) himalayana (Hodgson, 1841)	ZSIC, Naturalis
*Petauristaalbiventer* (Gray, 1834)	USNM
*Petauristaelegans* (Müller, 1840)	Naturalis, ZMA
*Petauristamagnificus* (Hodgson, 1836)	Naturalis
*Petauristanobilis* (Gray, 1842)	nil
*Petauristapetaurista* (Pallas, 1766)	Naturalis, ZMA
Petaurista*philippensis* (Elliot, 1839)	Naturalis, ZMBN
*Petauristamechukaensis* Choudhury, 2007	nil
*Petauristamishmiensis* Choudhury, 2009	nil
*Petinomysfuscocapillus* (Jerdon, 1847)	nil
*Ratufabicolor* (Sparrman, 1778)	Naturalis, ZMBN, RGM, LSUMZ
*Ratufaindica* (Erxleben, 1777)	ZMBN, Naturalis, ZMA
*Ratufamacroura* (Pennant, 1769)	nil
*Spermophilusfulvus* (Lichtenstein, 1823)	AMNH, KU, FMNH, MCZ, UMZM, ZFMK, RBINS, SMF, NHMUK, ISEZ-PAS, USNM, MVZ, MNHN
*Spermophilopsisleptodactylus* (Lichtenstein, 1823)	ROM, AMNH, KU, FMNH, MCZ, UMZM
*Tamiopsmcclellandii* (Horsfield, 1840)	Naturalis, ZMA, KIZ

**Legend**: AMNH American Museum of Natural History; ASNHC Angelo State Natural History Collections; BNHS Bombay Natural History Society; BPBM Bernice Pauahi Bishop Museum; BYU Brigham Young University (BYU) Life Science Museum; CAS California Academy of Sciences; FMNH Field Museum of Natural History; ISEZ-PAS Institute of Systematics and Evolution of Animals, Polish Academy of Sciences; KIZ Kunming Institute of Zoology, Chinese Academy of Sciences; KU University of Kansas, Biodiversity Institute and Natural History Museum; LACM Natural History Museum of Los Angeles County; LSUMZ Louisiana State University, Museum of Zoology; MACN Museo Argentino de Ciencias Naturales Bernardino Rivadavia; MCZ Harvard University, Museum of Comparative Zoology; MHNG Muséum d'Histoire Naturelle de la Ville de Genève; MNHN National Museum of Natural History, Paris, MVZ Museum of Vertebrate Zoology, University of California Berkeley, MZLU/LUND Lund University Biological Museum; Naturalis Naturalis Biodiversity Center; NHMO/UiO University of Oslo (UiO); NHMUK Natural History Museum, London; NMBT Natuurmuseum Brabant; NML World Museum, National Museums Liverpool; NMR Natural History Museum Rotterdam; NSMT National Science Museum, Tokyo; NSMT National Science Museum (Natural History); PSM Puget Sound Museum of Natural History, University of Puget Sound; RBINS Royal Belgian Institute of Natural Sciences; RGM Rijksmuseum van Geologie en Mineralogie (Naturalis); ROM Royal Ontario Museum; SMF Naturmuseum Senckenberg, Frankfurt; SNSB-SAPM SNSB-Staatssammlung für Anthropologie und Paläoanatomie München; UAM University of Alaska Museum; UF Florida Museum of Natural History- Zoology, Paleontology & Paleobotany; UMZM University of Michigan Museum of Zoology; USNM Smithsonian Institution, National Museum of Natural History; ZSIC Zoological Survey of India
