# Supplementary material for: A comprehensive database of squirrel distribution and occurrence in South Asia
Source: Biodivers Data J. 2023 Oct 27;11:e109946. doi: 10.3897/BDJ.11.e109946 (PMC10838086; doi:10.3897/BDJ.11.e109946)
Supplement: Supplementary material 1 — List of literature data from which occurrence data has been included in the database [file bdj-11-e109946-s001.pdf]

Supplementary Table 1: List of literature data from which occurrence data has been included in the database

Accepted taxonomy by GBIF Backbone	References
Belomys pearsonii (Gray, 1842)	[1–14]
Biswamoyopterus biswasi (Saha, 1981)	[1–14]
Callosciurus erythraeus (Pallas, 1779)	[1–17]
Callosciurus pygerythrus (I. Geoffroy Saint-Hilaire, 1833)	[1–15,18–20]
Dremomys lokriah (Hodgson, 1836)	[1–14,21,22]
Dremomys pernyi (Milne-Edwards, 1867)	[1–14]
Dremomys rufigenis (Blanford, 1878)	[1–14,23]
Eoglaucomys fimbriatus (Gray, 1837)	[1–14,24]
Eupetaurus cinereus (Thomas, 1888)	[1–14]
Funambulus layardi (Blyth,1849)	[1–14,25,26]
Funambulus obscurus (Pelzeln & Kohl, 1886)	[1–14,25]
Funambulus (Funambulus) palmarum (Linnaeus, 1766)	[1–14,27–71]
Funambulus (Prasadsciurus) pennantii (Wroughton, 1905)	[1–14,23,27–30,33,34,36–41,44,53,65,68,72–92]
Funambulus (Funambulus) sublineatus (Waterhouse, 1838)	[1–14,25,26,93,94]

Funambulus (Funambulus) tristriatus (Waterhouse, 1837)	[1–14,26,39,40,54,74,95–99]
Hylopetes alboniger (Hodgson, 1836)	[1–14]
Hylopetes phayrei (Blyth, 1859)	[1–14]
Marmota (Marmota) caudata (Geoffroy, 1844)	[1–14]
Marmota (Marmota) himalayana (Hodgson, 1841)	[1–14,21]
Petaurista petaurista albiventer (Gray, 1834)	[1–14,19,23,100]
Petaurista elegans (Müller, 1840)	[1–14,100]
Petaurista magnificus (Hodgson, 1836)	[1–14,23,100]
Petaurista nobilis (Gray, 1842)	[1–14,101]
Petaurista petaurista (Pallas, 1766)	[1–14,54,63,100,102,103]
Petaurista philippensis (Elliot, 1839)	[1–14,66,100,104–106]
Petaurista mechukaensis (Choudhury, 2007)	[1–14,100]
Petaurista mishmiensis (Choudhury, 2009)	[1–14,100]
Petinomys fuscocapillus (Jerdon, 1847)	[1–14,102,107]
Ratufa bicolor (Sparrman, 1778)	[1–14,20,108–124]
Ratufa indica (Erxleben, 1777)	[1–14,50,54,63,66,71,94,106,108,125

	–157]
Ratufa macroura (Pennant, 1769)	[1–14,58,63,93,100,106,108,158–168]
Spermophilus fulvus (Lichtenstein, 1823)	[1–14,169–173]
Spermophilopsis leptodactylus (Lichtenstein, 1823)	[1–14]
Tamiops mcclellandii (Horsfield, 1840)	[1–14,121,122]

1. Bombay Natural History Society. The journal of the Bombay Natural History Society. vol. 28. London, England: Forgotten Books; 1921.
2. Bombay Natural History Society. The journal of the Bombay Natural History Society. vol. 32 Bombay: Bombay Natural History Society; 1927.
3. Millard WS. The journal of the Bombay Natural History Society, vol. 24. London, England: Bombay Natural History Society; 1915.
4. Bombay Natural History Society. The journal of the Bombay Natural History Society, vol 21. Bombay Natural History Society; 1912.
5. Bombay Natural History Society. The journal of the Bombay Natural History Society, vol 22. Legare Street Press; 1913.
6. Bombay Natural History Society. Scientific results of the Mammal Survey. No. XLVI. Further examination of Singhalese species of Funambulus and description of a new subspecies. J Bombay Nat Hist Soc. 1926;31: 239–240.
7. Moore JC, Tate GHH. A study of the diurnal squirrels, Sciurinae, of the Indian and Indo-chinese subregions. Fieldiana. 1965;48: 1–351.
8. Zoological Survey of India. Records of the Indian Museum. Calcutta Pub. by order of the trustees of the Indian Museum 1907-1962; 1918. p. 460.
9. Ellerman JR, Morrison-Scott TCS. Checklist of Palaearctic and Indian mammals 1758 to 1946. British Museum of Natural History, London, 1966; 1966. p. 830.
10. Ellerman JR, Hayman RW, Holt GWC. The families and genera of living rodents. British Museum of Natural History, London, 1940; 1940. p. 728.

11. Floyd GF, Stojanovich CJ. The sucking lice. San Francisco, 1951; 1951. p. 336.

12. National Museum of Sri Lanka, National Museums of Ceylon, Colombo Museum. *Spolia zeylanica*. Colombo, Colombo Museum,; 1914. p. 468.

13. Giraud ED, Field Columbian Museum. A catalogue of the collection of mammals in the Field Columbian Museum. Chicago, Field Columbian Museum, 1907; 1907. p. 714.

14. Hayssen V. Patterns of body and tail length and body mass in Sciuridae. J Mammal. 2008;89: 852–873.

15. Chakraborty S. Studies on the Genus Callosciurus Gray (Rodentia--Sciuridae). Zoological Survey of India; 1985.

16. On the generic position of the groups of Squirrels typified by “Seiurus” berdmorei und pernyi respectively, with descriptions of some new Oriental species. J Bombay Nat Hist Soc. 1908;18: 244–249.

17. Keguo H, Kai L, Jing S, Jingqiu R, Jia Z, Yun Y, et al. Diversity of birds and mammals in Laojunshan region, Wenshan National Nature Reserve based on infrared cameras. Shou Lei Xue Bao. 2023;43: 89.

18. Sur S, Saikia PK, Saikia MK. Speed thrills but kills: A case study on seasonal variation in roadkill mortality on National Highway 715 (new) in Kaziranga-Karbi Anglong Landscape, Assam, India. NC. 2022;47: 87–104.

19. Millard WS. The journal of the Bombay natural history society, 1906-1907, vol. 17. London, England: Bombay Natural History Society; 1907.

20. Bhatt U, Adhikari BS, Lyngdoh S. Monitoring diversity and abundance of mammals with camera-traps: a case study of Manas National Park, Assam, India. CheckList. 2022;18: 1023–1043.

21. Katuwal H, Khanal B, Basnet K, Rai B, Devkota S, Rai S, et al. The mammalian fauna from the Central Himalayas, Nepal. Asian Journal of Conservation Biology. 08 2013;2: 21–29.

22. Chatterjee P, Bhattacharyya K, Bhattacharyya S. First record of the Long-snouted Bhutan Squirrel *Dremomys lokriah bhotia* (Wroughton, 1916) (Mammalia: Rodentia) from the state of West Bengal, India. Journal of Animal Diversity. 2022;4: 52–57.

23. Millard WS. The journal of the Bombay Natural History Society, vol. 23. London, England: Forgotten Books; 1914.

24. Pasha MKS, Suhail I. Range extension of the Kashmir flying squirrel (*Hylopetes fimbriatus* Gray). J Bombay Nat Hist Soc. 1997;94: 395–396.

25. Dissanayake R, Oshida T. The systematics of the dusky striped squirrel, *Funambulus sublineatus* (Waterhouse, 1838) (Rodentia: Sciuridae) and its relationships to Layard’s squirrel, *Funambulus layardi* Blyth, 1849. J Nat Hist. 2012;46: 91–116.

26. Robin VV, Davidar P. The vertical stratification of birds in mixed species flocks at Parambikulam, South India: a comparison between two habitats. J Bombay Nat Hist Soc. 2002;99: 389–399.

27. Zoological Survey of India. Vertebrate fauna of Kangerghati, Guru Ghasidas, and Sanjay National Parks (Madhya Pradesh and Chhatisgarh). Zoological Survey of India; 2008.

28. Ramakrishan, Chandra K, Nema DK, Ahirwar SC, Alfred JRB. Faunal Resources of National Parks of Madhya Pradesh and Chhattisgarh. Zoological Survey of India; 07 2006.

29. Harshey DK, Chandra K. Mammals of Madhya Pradesh and Chattisgarh. Zoos Print J. 11 2001;16: 659–668.

30. Zoological Survey of India. Fauna of Lonar Wildlife Sanctuary. Zoological Survey of India; 2008.

31. Ghosh AK, Zoological Survey of India. Fauna of Indravati Tiger Reserve, Madhya Pradesh. Zoological Survey of India; 1995.

32. Aditya V, Ganesh T. Mammals of Papikonda Hills, northern Eastern Ghats, India. J Threat Taxa. 10 2017;9: 10823.

33. Effect of landscape change on mammals in Eastern Ghats, India. In: Conservation Leadership Programme [Internet]. 15 Dec 2014. https://www.conservationleadershipprogramme.org/project/mammals-eastern-ghats-india/

34. Srinivasulu C, Nagulu V. Mammalian and avian diversity of the Nallamala Hills, Andhra Pradesh. Zoos Print J. 12 2001;17. doi:10.11609/JoTT.ZPJ.17.1.675-84

35. Khan YDI, Nautiyal S, Venkatesha MG. Mammalian Fauna of Semi-Arid Chitradurga District, Karnataka, India. Environ Ecol. 2020. Available: https://www.researchgate.net/profile/Melally-Venkatesha/publication/348565277_Mammalian_Fauna_of_Semi-Arid_Chitradurga_District_Karnataka_India/links/6005539645851553a050f63b/Mammalian-Fauna-of-Semi-Arid-Chitradurga-District-Karnataka-India.pdf

36. Zoological Survey of India. Fauna of Bihar, Including Jharkhand. Zoological Survey of India; 2004.

37. Zoological Survey of India. Fauna of Orissa. Zoological Survey of India; 1993.

38. Zoological Survey of India. Fauna of Andhra Pradesh. Zoological Survey of India; 2004.

39. Zoological Survey of India. Fauna of Maharashtra. Zoological Survey of India; 2012.

40. Zoological Survey of India. Fauna of Karnataka. Zoological Survey of India; 2013.

41. Zoological Survey of India. Fauna of West Bengal. Zoological Survey of India; 1993.

42. Thirumalai G, Krishnan S, Zoological Survey of India. Fauna of Biligiri Rangaswamy Temple Wildlife Sanctuary, Karnataka. Zoological Survey of India; 2006.

43. Thirumalai G, Krishnan S, of India ZS. Fauna of Bannerghatta National Park. Zoological Survey of India; 2007.

44. Balasubramanian P. Some notes on the fruits, seeds and nectar consumed by Three-striped palm squirrel *Funambulus palmarum* at Point Calimere Wildlife Sanctuary, Tamil Nadu. J Bombay Nat Hist Soc. 1995;92: 256–258.

45. Johnsingh AJT. Some Aspects of the Ecology and Behavior of the Indian Fox *Vulpes bengalensis*. J Bombay Nat Hist Soc. 1978;75: 397–405.

46. Bhardwaj GS, Habib B, Nigam P, Sengupta D, Kari B. Camera trapping data indicates temporal niche segregation among mammals in a tropical deciduous forest. SRLS. 2022;3: 8–26.

47. Konhar P, Sahoo BK. Monitor and assess the total damage caused by rodents in various coconut orchards of coastal Odisha. Pharma Innovation. 2022;11: 2099–2102.

48. Kumar P, Ojha AK. Some aspects of feeding ecology and behavior of House crow (*Corvus splendens*) in an urban habitat of city Prayagraj (U.P.), India. J App Biol Biotech. 2022;11: 45–50.

49. Ahsan MM. Study on Diversity of Rodents (Mammalia : Rodentia) in and around Achalpur City, District Amravati, Maharashtra, India. International Journal of Scientific Research in Biological Sciences. 2022;9: 35–38.

50. Nature Science Foundation. Technical report of green campus audit Dr. R.A.N.M. Arts & Science College, Chennimalai RD, Rangapalayam, Erode – 638009, Tamil Nadu, India. 2019.

51. Prakash I. Palm Squirrel (*Funambulus palmarum*, Linnaeus) in Australia. J Bombay Nat Hist Soc. 1967;64: 108–108.

52. Sadakathulla S, Abdul Kareem A. Cannibalism in south Indian palm squirrel *Funambulus palmarum*. J Bombay Nat Hist Soc. 1995;92: 113–114.

53. Sharma SK. A note on the latex licking habit of Five striped and Three striped Palm Squirrels. J Bombay Nat Hist Soc. 2007;104: 82–83.

54. Bombay Natural History Society. The journal of the Bombay Natural History Society. Bombay, Bombay Natural History Society; 2011. p. 516.

55. Berghaier R. Observations on Eco-tourism and Conservation in India. Animal keepers' forum. 2012;39: 268–276.

56. Sivakumaran N, Rahmani AR. Spotted Dove *Streptopelia Chinensis* feeding on winged termites. J Bombay Nat Hist Soc. 2005;102: 115–115.

57. Chauhan NS. A note on the post-partum reproduction in the Short-tailed Bandicoot rat *Nesokia indica*. J Bombay Nat Hist Soc. 1987;84: 426–427.

58. Davidar P. Grizzled giant squirrel *Ratufa macroura* distribution in Kudirayar, India. J Bombay Nat Hist Soc. 1989;86: 437–437.

59. Johnsingh AJT, Paramanandham K, Murali S. Foraging behavior and interactions of White-headed babblers *Turdoides affinis* with other species. J Bombay Nat Hist Soc. 1982;79: 503–514.

60. Patton WS, Cragg FW. A textbook of medical entomology. London, Christian Literature Society for India, 1913; 1913. p. 1146.

61. Thomas Oldfield. Scientific results from the Mammal Survey. No. XI. J Bombay Nat Hist Soc. 1915;24: 29–65.

62. Wroughton RC, Ryley KV. Scientific results from the mammal Survey. Part III. J Bombay Nat Hist Soc. 1913;22: 13–21.

63. Easa P. Prey predator studies in Eravikulam National Park. 1995 [cited 8 Jun 2023]. Available: https://www.semanticscholar.org/paper/87ec26c32e320e3caa1a76f798e4dbddbaf3cd9c

64. Gamble JS, Asiatic Society of Bengal, Asiatic Society (Kolkata, India). Journal and proceedings of the Asiatic Society of Bengal. Calcutta, Asiatic Society of Bengal,; 1914. p. 660.

65. Indian Museum., Zoological Survey of India. Records of the Indian Museum. Calcutta: Pub. by order of the trustees of the Indian Museum; 1913.

66. Pradhan MS. Qualitative analysis of major vertebrate fauna from Wardha river basin (Maharashtra State). J Bombay Nat Hist Soc. 1997;94: 71–103.

67. Crimmins ML. Treatment of Snake bite. J Bombay Nat Hist Soc. 1932;35: 690–690.

68. Wroughton RC. The common Palm-Squirrel. J Bombay Nat Hist Soc. 1905;16: 406–413.

69. Bombay Natural History Society. The journal of the Bombay Natural History Society, vol 84. General Books; 1987.

70. Patton. A textbook of medical entomology. General Books; 2010.

71. Ali R. Feeding ecology of the Bonnet Macaque at the Mundanthurai Sanctuary, Tamil Nadu, India. J Bombay Nat Hist Soc. 1986;83: 98–110.

72. of India ZS. Fauna of Valmiki Tiger Reserve. Zoological Survey of India; 1998.

73. of India ZS. Some Selected Fauna of Asola-Bhatti Wildlife Sancturary, [Delhi]. Zoological Survey of India; 2003.

74. Zoological Survey of India. Fauna of Goa. Zoological Survey of India; 2008.

75. Zoological Survey of India. Fauna of Kudremukh National Park (Karnataka). Zoological Survey of India; 2007.

76. Parasara UA, Parasharya BM, Mathew KL. Five-striped squirrel *Funambulus pennanti* Wroughton, a predator of *Helicoverpa armigera* Hb. (Lepidoptera: Noctuidae). J Bombay Nat Hist Soc. 1997;94: 562–564.

77. Naik SAN. Five striped squirrel *Funambulus pennantii* observed eating honey. J Bombay Nat Hist Soc. 1998;95: 500–500.

78. Sharma SK. Use of palm fronds for nest sites by the Northern palm squirrel *Funambulus pennantii* Wroughton. J Bombay Nat Hist Soc. 2012;109: 197–198.

79. Meena SS, Koli VK, Bhatnagar C, Sharma SK. Predation on Northern house Gecko *Hemidactylus flaviviridis* Ruppell by female Northern palm squirrel *Funambulus pennantii* Wroughton. J Bombay Nat Hist Soc. 2012;109: 198–199.

80. Harit DN. Unusual feeding behaviour of squirrel, *Funambulus* spp. J Bombay Nat Hist Soc. 1996;93: 84–84.

81. Mukherjee AK. The Sundarban of India and Its Biota. J Bombay Nat Hist Soc. 1975;72: 1–20.

82. Manohar BR, Rajasekaran M. Additional record on mortality from hailstorm at Jaipur, India. J Bombay Nat Hist Soc. 1989;86: 461–461.

83. Sandhu PS, Kapoor VC. New record of acarine ectoparasites on the Northern palm squirrel, *Funambulus pennanti* Wroughton from India. Entomol News. 1976;87: 292–294.

84. Millard WS. The journal of the Bombay natural history society, vol. 22. London, England: Bombay Natural History Society; 1913.

85. Millard WS. The journal of the Bombay natural history society, vol. 18. London, England: Bombay Natural History Society; 1907.

86. Rabari VM, Malik A, Dharaiya N. A preliminary study on relative abundance of wild mammals based on camera trap in Balaram-Ambaji Wildlife Sanctuary, Gujarat State, India. Journal of Animal Diversity. 2022;4: 53–61.

87. Acharya V, Charan PD, Chhangani AK. Avian fauna in the human landscape around Bikaner city, Rajasthan, India. Rajasthali Journal. July-September 2022;1: 47–60.

88. The value of an urban park in preserving a small number of wild mammals in ecopark, Kolkata, West Bengal, India. International Research Journal of Modernization in Engineering Technology and Science. 2022.

89. Biswas S, Bhowmik T, Ghosh K, Roy A, Lahiri A, Sarkar S, et al. Scavengers in the human-dominated landscape: an experimental study. arXiv [q-bio.PE]. 2022. Available: http://arxiv.org/abs/2208.05030

90. Yousefi M, Mahmoudi A, Kafash A, Khani A, Kryštufek B. Biogeography of rodents in Iran: species richness, elevational distribution and their environmental correlates. Mammalia. 2022;86: 309–320.

91. Chhangani AK. Mortality of Wild Animals in Road Accidents in Kumbhalgarh Wildlife Sanctuary, Rajasthan, India. J Bombay Nat Hist Soc. 2004;101: 151–154.

92. Lister MD. Some bird associations of Indian built up areas. J Bombay Nat Hist Soc. 1953;51: 369–377.

93. Dasanayake T, Mahaulpatha D. Influence of recreational trails on tetrapod vertebrates within Horton Plains National Park. In: ResearchGate [Internet]. 2019 [cited 8 Jun 2023]. Available: https://www.researchgate.net/profile/Tharanga-

Dasanayake/publication/339067977_Influence_of_recreational_trails_on_tetrapod_vertebrates_within_Horton_Plains_National_Park/links/5e3ba5c3a6fdccd9658aaa91/Influence-of-recreational-trails-on-tetrapod-vertebrates-within-Horton-Plains-National-Park.pdf

94. Shanker K. Small mammals in montane ecosystems of the Nilgiris, Southern India: their ecology and natural history. J Bombay Nat Hist Soc. 2003;100: 46–57.

95. Zoological Survey of India. Fauna of Eravikulam National Park. Zoological Survey of India; 2002.

96. Sakthivel P, Neelanarayanan P. Burrow structure of Indian bush rat *Golunda ellioti* and Brown spiny mouse *Mus Platythrix* in Tiruchirappalli District, Tamil Nadu. J Bombay Nat Hist Soc. 2008;105: 329–332.

97. Thejaswi S. Kemmangundi revisited: Notes on birds observed at the Bababudan Hills, Karnataka, South India. J Bombay Nat Hist Soc. 2004;101: 235–243.

98. Bhat SK. An instance of fostering in captivity in the Western Ghats squirrel Funambulus tristriatus. J Bombay Nat Hist Soc. 1982;79: 407–408.

99. Bombay Natural History Society. The journal of the Bombay Natural History Society; vol.105. Legare Street Press; 2008.

100. Flower SS. On the Mammalia of Siam and the Malay Peninsula. Proc Zool Soc Lond. 1900;1900: 306–379.

101. Choudhury Anwaruddin. *Petaurista nobilis singhei*: First Record in India and a Note on Its Taxonomy. J Bombay Nat Hist Soc. 2002;99: 30–34.

102. Bombay Natural History Society. Journal of the Bombay Natural History Society, vol 90. Legare Street Press; 1993.

103. Suis MAF, Miun J, Tingkoi L, Hastie AYL, Chyang ACY, Nilus R. A Demi-Decade of Mammal Research: A Rapid Assessment within the Heart of Borneo in Sabah. Trop Life Sci Res. 2023;34: 261–277.

104. Bombay Natural History Society. The journal of the Bombay Natural History Society, vol 48, 1948. General Books; 1948.

105. Phillips WWA. Rearing a baby Ceylon Grey flying squirrel (*Petaurista philippensis lanka*). J Bombay Nat Hist Soc. 1951;50: 164–165.

106. Naresh B, Sankari A, Baskaran N, Vaidyula VR, Saravanan M. Population density of Indian Giant Squirrel (*Ratufa indica*) in Srivilliputhur Grizzled Giant Squirrel Wildlife Sanctuary, Tamil Nadu. International Journal of Multidisciplinary Research and Development 2014; 1(5): 37-41. 01 2014;1: 37–41.

107. Kannan R, James DA. Breeding biology of the great pied hornbill (*Buceros bicornis*) in the Anaimalai hills of southern India. J Bombay Nat Hist Soc. 1997;94: 451–465.

108. Bahuguna A, Singh A. Molecular characterization by using 12SrRNA and Cytochrome b for identification of species of genus Ratufa (Rodentia: Sciuridae) including *Ratufa indica*, endemic species of India. Mitochondrial DNA B Resour. 2019;4: 3085–3091.

109. Albert-Daviaud A, McConkey KR, Jha N, Fontaine C, Kitamura S, Nathalang A, et al. Threatened species are disproportionately important interactors in a seed dispersal network in Southeast Asia. Integrative Conservation. 2022;1: 25–39.

110. Kakati R, Borah D, Saikia PK, Hazarika A. Birds of Behali Wildlife Sanctuary, an Important Bird Area of Assam, India. AUPCStudiaNaturae. 2022; 77–107.

111. Babu P, Gupta S, Sethy J, Ahmed MF, Chatakonda MK. Observations of courtship and mating behaviour of Malayan giant squirrel (*Ratufa bicolor*, Sparrman, 1778) in wild. Proc Zool Soc Lond. 2022;75: 387–390.

112. Kong L, Wang W, Cong H, Liu Z, Li Y. Complete mitochondrial genome of the black giant squirrel *Ratufa bicolor* (Rodentia: Sciuridae). Mitochondrial DNA. 2015;26: 759–760.

113. Sengupta S, Singha H, Deb P. Ground foraging behaviour of Malayan giant squirrel (*Ratufa bicolor*). Curr Sci. 2016;110: 2223–2225.

114. Singh R, Alves R, Ralen O. Hunting of kebung (*Ratufa bicolor*) and other squirrel species from Morang forest by the Adi tribe of Arunachal Pradesh, India: Biocultural conservation and livelihood dimensions. Regional Environ Change. 08 2014;14: 1479–1490.

115. Chakraborty S, Chakraborty R. Field observations on the Malayan giant squirrel, *Ratufa bicolor gigantea* (M'Clelland) and some other diurnal squirrels of Jalpaiguri district, west Bengal. Rec Zool Surv India. 1991;88: 195.

116. Chatterjee P, Tripathy B, Chandra K, Saha GK, Mondal K. Climate Change Alarms the Survival of Near Threatened Species Malayan Giant Squirrel (*Ratufa bicolor* Sparrman, 1778) in India. Jmam. 2020;45: 289–302.

117. Jansen J. *Notoedres centrifera* n. sp. from the squirrel *Ratufa bicolor*. Natuurtijdschriften.nl [Internet]. Available: https://natuurtijdschriften.nl/pub/1014397/EB1963023012007.pdf

118. Dobroruka LJ. Notes on the behaviour of the Malayan giant squirrel. Int Zoo Yearbook. 1975;15: 207–212.

119. Duc NM, Trang LQ, Tam NM. Assessing species diversity of rodents (Rodentia) in Hoang. 2017 [cited 8 Jun 2023]. Available: https://www.semanticscholar.org/paper/9257be02377c2dc51fb2d38498525df0cd91dfd4

120. Wahyuni AI, Khairiah A, Mulyawan B. Arboreal mammals inventory in Tapos area of gunung Gede Pangrango national park. Bioscience. 2022;6: 72–78.

121. Brockelman WY, McConkey KR, Nathalang A, Somnnuk R, Santon J, Matmoon U. Dispersal success of a specialized tropical tree depends on complex interactions among diverse mammalian frugivores. Global Ecology and Conservation. 2022;40: e02312.

122. Saikia U, Meetei AB. Diversity, distribution, and abundance status of small mammalian fauna (Chiroptera: Rodentia: Eulipotyphla) of Manipur, India. J Threat Taxa. 2022;14: 21751–21768.

123. Bujarbarua P, Chetry D, Das J, Sarma SK, Bhattacharjee PC. A Note on Some Food Plants of the Malayan Giant Squirrel *Ratufa bicolor* in Gibbon Wildlife Sanctuary, Jorhat, Assam. J Bombay Nat Hist Soc. 2001;98: 101–102.

124. Choudhury Anwaruddin. Red panda *Ailurus fulgens* F. Cuvier in the north-east with an important record from Garo Hills. J Bombay Nat Hist Soc. 1997;94: 145–147.

125. Karanth KU. Bhadra Wildlife Sanctuary, India and its endangered ecosystem. J Bombay Nat Hist Soc. 1982;79: 79–86.

126. Mehta P. Leopard (Panthera pardus) attempting to prey on Indian giant squirrel (*Ratufa indica centralis*). J Bombay Nat Hist Soc. 1997;94: 555–556.

127. Worah S, Bharucha EK, Rodgers WA. The use of Geographic Information Systems in identifying potential wildlife habitat. J Bombay Nat Hist Soc. 1989;86: 125–128.

128. Karr JR. Ecological and behavioral notes on the Lion tailed Macaque *Macaca silenus* in South India. J Bombay Nat Hist Soc. 1973;70: 191–193.

129. Borges Renee Maria. Possible play between the Indian giant squirrel *Ratufa indica* indica and the Common Langur *Presbytis entellus*. J Bombay Nat Hist Soc. 1986;83: 197–197.

130. Abdulali Humayun, Daniel JC. Races of the Indian Giant Squirrel (*Ratufa indica*). J Bombay Nat Hist Soc. 1952;50: 469–474.

131. Datta A. Anti-predatory response of the Indian giant squirrel *Ratufa indica* to predation attempts by the crested hawk eagle *Spizaetus cirrhatus limnaetus*. J Bombay Nat Hist Soc. 1998;95: 332–335.

132. Datta A. Daytime resting in the nest: An adaptation by the Indian Giant Squirrel *Ratufa indica* to avoid predation. J Bombay Nat Hist Soc. 1999;96: 132–134.

133. Das UK, Samantarai R, Tyagi V, Panth S. Population density, nesting ecology and conservation of the Indian giant squirrel (*Ratufa indica*) Erxleben, 1777 in protected areas of Odisha. 02 2022.

134. Palei N, Palei HS, Rath B, Mishra A. Fodder plants of Indian giant squirrel (*Ratufa indica*) in Kapilash Wildlife Sanctuary, Odisha, India. e-planet. 12 2017;15: 155–160.

135. Prakash S, Mishra AK, Raziuddin M. Studies on the nesting habits of Indian Giant Squirrel *Ratufa indica centralis* Ryley 1913 in Dalma Wildlife Sanctuary, Jharkhand, India. Columban journal of Life Sciences. 12 2011;12: 9–18.

136. Dubey S, Malviya S, Pant H. Bird Composition of Pachmarhi Biosphere Reserve, Madhya Pradesh. 03 2022. p. 26.

137. Baskaran N, Venkatesan S, Mani J, Srivastava SK, Desai AA. Some aspects of the ecology of the Indian Giant Squirrel *Ratufa indica* (Erxleben, 1777) in the tropical forests of Mudumalai Wildlife Sanctuary, southern India and their conservation implications. J Threat Taxa. 2011;3: 1899–1908.

138. Arockianathan S. Nesting behavior of Indian giant squirrel (*Ratufa indica* Erxleben, 1777) in Mudumalai Tiger Reserve, western ghats, southern India. Rodents. IntechOpen; 2021.

139. Pradhan A, Shrotriya S, Rout S, Dash P. Nesting and feeding habits of the Indian giant squirrel (*Ratufa indica*) in Karlapat wildlife sanctuary, India. Anim Biodivers Conserv. 01 2017;40: 63–69.

140. Nayak BK, Patra AK. Feeding and nesting ecology of Indian giant squirrel *Ratufa indica* (Erxleben,1777) in Kuldiha wildlife sanctuary, Balasore, Odisha, India and its conservation. International Journal of Bioassays. 2015. Available: https://www.academia.edu/12001793/Feeding_and_nesting_ecology_of_Indian_giant_squirrel_Ratufa_indica_erxleben_1777_in_Kuldiha_wildlife_sanctuary_Balasore_Odisha_India_and_its_conservation

141. Borges RM. Sexual and site differences in calcium consumption by the Malabar Giant Squirrel *Ratufa indica*. Oecologia. 1990;85: 80–86.

142. Jathanna D, Kumar NS, Karanth KU. Measuring Indian giant squirrel (*Ratufa indica*) abundance in southern India using distance sampling. Curr Sci. 2008;95: 885–888.

143. Mishra AT, Kazmi SEH, Prakash S. Nesting and Feeding Behaviour of Indian Giant Squirrel (*Ratufa indica*) in Dalma Wildlife Sanctuary, Jamshedpur (Jharkhand). Indian For. 2011;137: 1155–1159.

144. Somanathan H, Mali S, Borges R. Arboreal larder-hoarding in the tropical Indian giant squirrel *Ratufa indica*. Ecoscience. 06 2007;14: 165–169.

145. Palei HS, Sahu HK, Nayak AK. Population Density, Diurnal Activity Pattern and Food Preference of Indian Giant Squirrel *Ratufa indica* in Similipal Tiger Reserve, Eastern India. jmam. 2015;40: 257–263.

146. Sayyed A. Albinism in Indian Giant Squirrel, *Ratufa indica* (Erxleben) and Lesser Bandicoot Rat, *Bandicota bengalensis* (Gray) (Rodentia: Mammalia) from Satara District, Maharashtra. Small Mammal Mail. 2014 [cited 8 Jun 2023]. Available: https://www.academia.edu/7095282/Albinism_in_Indian_Giant_Squirrel_Ratufa_indica_Erxleben_and_Lesser_Bandicoot_Rat_Bandicota_bengalensis_Gray_Rodentia_Mammalia_from_Satara_District_Maharashtra

147. Kumbhar, Kumbhar A, Pradhan A, Patwardhan G. Open Access Short Communication Some Observation on Dray Building and Jumping Behavior of Indian Giant Squirrel *Ratufa indica* (Erxleben, 1777). Universal Journal of Environmental Research and Technology. 08 2012;2: 366–368.

148. Pillai R, Kikani J, Walmiki N, Parab U, Karangutkar S. A record of *Python molurus* (Linnaeus, 1758) feeding on Ratufa indica Erxleben. HNO. 2020;13: 389–390.

149. Adhikary CC, Ghosh AK. On the Occurrence of *Linognathus vituli* (Linnaeus) (Anoplura : Linognathidae) on a Giant Squirrel, *Ratufa indica* from North Bengal. Records of the Zoological Survey of India. 1993;93: 317–319.

150. Ragothaman V, Murali N, Chirukandoth S, Manokari K. Karyotype of Indian Giant Squirrel (*Ratufa indica*). Curr Sci. 03 2016;110: 983–985.

151. Shukla PN, Mishra VK. Population distribution of Indian Giant squirrel *Ratufa indica* in dry and moist deciduous forest of Sironcha forest Division, Central India. Indian For. 2017;143: 1021–1026.

152. Kanoje RS. Nesting sites of Indian giant squirrels in Sitanadi Wildlife Sanctuary, India. Curr Sci. 2008;95: 882–884.

153. Samson A. Occupation of Indian Giant Squirrel nests by White-rumped Vultures *Gyps bengalensis* in India. Podoces. 01 2015;2015: 35–36.

154. Supriya Devi R. Preferred Food plants of *Ratufa indica* (Erxleben, 1777): A fragmented species of Rodentia. Journal of biodiversity and conservation. 03 2020;4: 277–281.

155. Borges RM. Figs, Malabar Giant Squirrels, and Fruit Shortages Within Two Tropical Indian Forests. Biotropica. 1993;25: 183–190.

156. Oates 1944- John F. The Status of the South Indian Black Leaf Monkey *Presbytis johnii* in the Palni Hills India. J Bombay Nat Hist Soc. 1978;75: 1–12.

157. Ramachandran KK, Joseph GK. Distribution and demography of diurnal primates in Silent Valley National Park and adjacent areas, Kerala, India. J Bombay Nat Hist Soc. 2001;98: 191–196.

158. Sharma N. Colour variation in populations of the grizzled giant squirrel *Ratufa macroura*. J Bombay Nat Hist Soc. 1997;94: 565–565.

159. Sathasivam K, Santharam V, Sudhakar KV, Narayanan TB. An unreported population of the Grizzled giant squirrel *Ratufa macroura*. J Bombay Nat Hist Soc. 2008;105: 213–213.

160. Karthikeyan S, Prasad JN, Arun B. Grizzled giant squirrel *Ratufa macroura* Thomas and Wroughton at Cauvery Valley in Karnataka. J Bombay Nat Hist Soc. 1992;89: 360–361.

161. Vanitharani J. Conservation status and guidelines for the maintenance of endangered Grizzled Giant Squirrel *Ratufa macroura* in Srivilliputhur Wildlife Sanctuary. In: Sivaperuman C, Venkataraman K, editors. Indian Hotspots: Vertebrate faunal diversity, Conservation and management, Volume 2. Singapore: Springer Singapore; 2018. pp. 297–307.

162. Thomas K. Alarming population status of the Grizzled Giant Squirrel *Ratufa macroura* (Mammalia: Rodentia: Sciuridae) in Chinnar Wildlife Sanctuary, the Western Ghats, India. J Threat Taxa. 2018;10: 12350–12356.

163. Thomas K. Characterisation of breeding habitat of Grizzled Giant Squirrel *Ratufa macroura* (Mammalia: Sciuridae) in Chinnar Wildlife Sanctuary, Western Ghats, India. J Threat Taxa. 2021;13: 18993–19001.

164. Joshua J, Johnsingh AJT. Impact of biotic disturbances on the habitat and population of the endangered grizzled giant squirrel *Ratufa macroura* in south India. Biol Conserv. 1994;68: 29–34.

165. Vimalraj S, Raman K, Reddy DA, Harikrishnan B, Krishnakumar BM, Selvan KM. A new sight record and range extension of the Grizzled Giant Squirrel *Ratufa macroura dandolena* (Mammalia: Rodentia: Sciuridae) in the Eastern Ghats of southern peninsular India. J Threat Taxa. 2018;10: 11240–11242.

166. Baskaran N, Senthilkumar K, Saravanan M. A new site record of the Grizzled Giant Squirrel *Ratufa macroura* (Pennant, 1769) in the Hosur forest division, Eastern Ghats, India and its conservation significance. J Threat Taxa. 2011; 1837–1841.

167. Babu S, Kalaimani A. New site record of Grizzled Giant Squirrel *Ratufa macroura* from Thiruvannamalai Forest Division, Eastern Ghats, Tamil Nadu, India. J Threat Taxa. 2014;6: 5492–5493.

168. Thomas K, Vinodkumar DK, John JM, Shaji M, Nammer PO. A report on the possible interbreeding between Grizzled Giant Squirrel *Ratufa macroura* and Indian Giant Squirrel *Ratufa indica* from Chinnar Wildlife Sanctuary in the southern Western Ghats, India. J Threat Taxa. 2018;10: 13024–13028.

169. Matrosova VA, Ivanova AD, Volodina EV, Volodin IA, Alexandrov DY, Sibiryakova OV, et al. Phylogenetic relationship and variation of alarm call traits of populations of red-cheeked ground squirrels (*Spermophilus erythrogenys sensu lato*) suggest taxonomic delineation. Integr Zool. 2019;14: 341–353.

170. Matrosova VA, Schneiderová I, Volodin IA, Volodina EV. Species-specific and shared features in vocal repertoires of three Eurasian ground squirrels (genus *Spermophilus*). Acta Theriol . 2012;57: 65–78.

171. Vasilieva NA, Tchabovsky AV. Timing is the only thing: reproduction in female yellow ground squirrels (*Spermophilus fulvus*). Can J Zool. 2014;92: 737–747.

172. Vasilieva NA, Tchabovsky AV. Reproductive decisions in a “Fast-Living” Sciurid: A case study of the Yellow Ground Squirrel (*Spermophilus fulvus*). Biology Bulletin Reviews. 2018;8: 12–22.

173. Asgharzadeh A, Kaboli M, Rajabi-Maham H, Naderi M. Phylogeny and genetic structure of the Yellow ground squirrel, *Spermophilus fulvus* (Lichtenstein, 1823), in Iran. Mamm Biol. 2019;98: 137–145.
